# Unveiling the impact of interferon genes on the immune microenvironment of triple-negative breast cancer: identification of therapeutic targets

**DOI:** 10.3389/fbinf.2025.1629526

**Published:** 2025-10-08

**Authors:** Ying Liu, Jiayi Cai, Aamir Fahira, Kai Zhuang, Jiaojiao Wang, Zhi Zhang, Lin Yan, Yong Liu, Defang Ouyang, Zunnan Huang

**Affiliations:** 1 The Affiliated Dongguan Songshan Lake Central Hospital, Guangdong Medical University, Dongguan, China; 2 Guangdong Medical University Key Laboratory of Big Data Mining and Precision Drug Design, Dongguan Key Laboratory of Computer-Aided Drug Design, Guangdong Provincial Key Laboratory for Research and Development of Natural Drugs, School of Pharmacy, Guangdong Medical University, Dongguan, China; 3 The First Dongguan Affiliated Hospital, Guangdong Medical University, Dongguan, China; 4 State Key Laboratory of Quality Research in Chinese Medicine, Institute of Chinese Medical Sciences (ICMS), University of Macau, Macau, China

**Keywords:** triple-negative breast cancer, interferon gene, prognostic signature, ceRNA network, immune microenvironment

## Abstract

**Objective:**

Triple-negative breast cancer (TNBC), a classic subtype of breast cancer, is challenging to treat due to the lack of drug-targeting receptors. This study aims to explore interferon-related prognostic molecular biomarkers in TNBC and their potential competing endogenous RNA (ceRNA) regulatory network in TNBC.

**Methods:**

RNA expression profiles and interferon genes were downloaded from the Cancer Genome Atlas (TCGA) database and the Gene Set Enrichment Analysis (GSEA) website, respectively. Univariate and multivariate Cox regression analyses were performed to identify prognostic genes and construct a risk model. Single-sample GSEA (ssGSEA) and the CellMiner database were used to explore the relationships between prognostic genes and both tumor immune microenvironment and drug sensitivity, respectively. The lncRNA-miRNA-mRNA network associated with prognosis was constructed using the ENCORI database. Finally, the potential interferon-associated lncRNA/miRNA/mRNA regulatory axis was identified through correlation analysis. The abnormal expressions of prognostic genes were validated in three TNBC tumor cell lines compared to normal mammary epithelial cells by using quantitative real-time polymerase chain reaction (qRT-PCR).

**Results:**

The TNBC prognostic signature comprising four interferon genes (STXBP1, LAMP3, CD276, and POLR2F) was identified, with their expression significantly correlated with the infiltration abundance of multiple immune cells and the drug sensitivity of 30 diverse drugs (ARQ-680, Fluphenazine, and Chelerythrine, etc.). Furthermore, an interferon-related genes prognostic ceRNA network was further constructed, consisting of 248 lncRNAs, 66 miRNAs, and 4 mRNAs. As a result, 5 interferon-related ceRNA regulatory axes (AC124067.4/hsa-miR-455-3p/STXBP1, RBPMS-AS1/hsa-miR-455-3p/STXBP1, DNMBP-AS1/hsa-miR-455-3p/STXBP1, FAM198B-AS1/hsa-miR-455-3p/STXBP1, LIFR-AS1/hsa-miR-455-3p/STXBP1) associated with TNBC progression were identified. QRT-PCR results showed that all four prognostic mRNAs were upregulated in TNBC cells.

**Conclusion:**

This study established a prognostic signature and a ceRNA network associated with interferon in TNBC, and identified five key regulatory axes. In the prognostic signature and the ceRNA axes, STXBP1, RBPMS-AS1, and FAM198B-AS1 were first reported as potential biomarkers of TNBC. These findings have the potential to provide new insights into the mechanisms driving TNBC tumorigenesis and development.

## 1 Introduction

Breast cancer (BC) is among the most prevalent malignant tumors and the leading cause of cancer-related deaths among women globally (Bray et al., 2024; Kim et al., 2025). According to the latest report of the World Health Organization, by 2050, the number of new breast cancer cases worldwide is expected to increase by 38%, and the number of deaths due to breast cancer will increase by 68% (Kim et al., 2025). Triple-negative breast cancer (TNBC), a distinct subtype of BC, accounting for approximately 15%–20% of invasive breast cancer cases, is characterized by high heterogeneity, aggressiveness, and recurrence rates (Almansour, 2022). Additionally, TNBC lacks specific targets and effective targeted therapies, which is a major factor contributing to the failure of anti-cancer treatments and subsequent patient mortality. At present, surgery combined with systemic chemotherapy remains the standard treatment for TNBC. However, conventional postoperative adjuvant chemotherapy shows poor efficacy, with residual metastatic lesions often leading to tumor recurrence (Chaudhary et al., 2018). Thus, identifying new molecular biomarkers is crucial for the early diagnosis, prognosis, and monitoring of TNBC recurrence in patients.

As a multifunctional cytokine, Interferon (IFN) plays a key role in the antiviral activity, anti-proliferative, and immunomodulation (Sikora and Smedley, 1983). IFN can exert both direct and indirect anti-tumor effects by inducing apoptosis, blocking the cell cycle, and activating immunomodulatory function (Chang and Ho, 2025). For example, IFN-β signaling can inhibit the stemness of cancer cells in TNBC (Doherty et al., 2017). Moreover, the activation of IFN signaling is crucial for initiating anti-tumor immunity. Ligand-dependent corepressor protein (LCOR) binds to IFN-stimulated response elements (ISRE) in an IFN signaling transduction-independent manner to enhance the effectiveness of immune checkpoint blockade (ICB) in TNBC (Pérez-Núñez et al., 2022). IFN may influence the occurrence and progression of TNBC by modulating the immune microenvironment. However, the underlying mechanism of IFN’s role in TNBC immunomodulation remains unclear. Therefore, further research is needed to elucidate the relationship between IFN and immunity in TNBC.

Recent researches suggest that the lncRNA-miRNA-mRNA regulatory network plays a crucial role in the pathogenesis and progression of various cancers, including liver cancer (Shao et al., 2022), bladder cancer (Xiong et al., 2022), and other malignancies (Zhou et al., 2019). This regulatory network originated from the competitive endogenous RNA (ceRNA) proposed by Salmena et al. (2011). According to this theory, lncRNA competes with miRNA to regulate mRNA expression levels, thereby influencing protein translation and related cellular activities. Recently, the lncRNA-miRNA-mRNA regulatory axis has also been found to have a significant impact on the occurrence, development, and prognosis of TNBC. For instance, Yang et al. (2021) showed that lnc049808, acting as a ceRNA for miR-101, could upregulate FUNDC1, thereby promoting the proliferation, invasion, and metastasis of TNBC cells. Similarly, Li C. X. et al. (2022) revealed that knocking down lncLRP11-AS1 promoted the expression of miR-149-3p, leading to a decrease in NRP2 levels and inhibiting the malignant progression of TNBC. Although some progress has been made in studying the ceRNA network of TNBC (Ma et al., 2020), research on its interferon-related ceRNA regulatory axis remains unexplored. Therefore, exploring the IFN-related ceRNA network and identifying its key regulatory axes and targets may uncover novel therapeutic targets for TNBC treatment.

**FIGURE 1:**
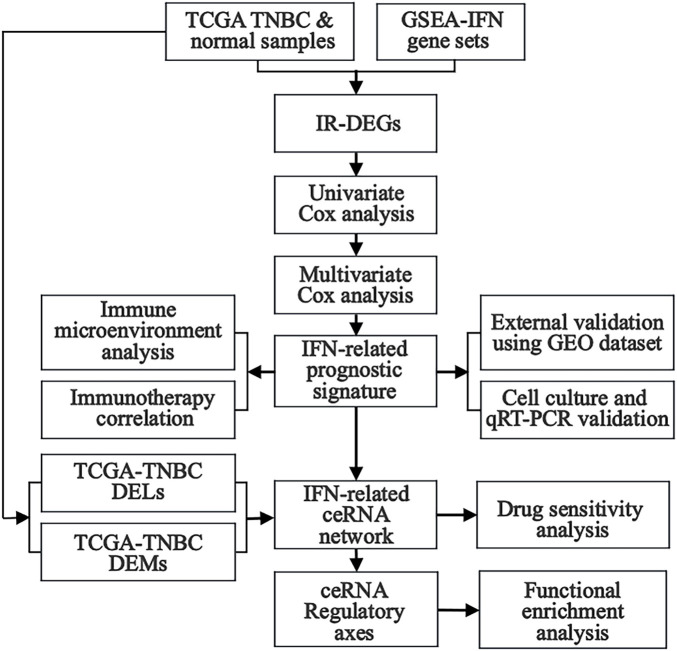
The flow diagram illustrates the step-by-step analysis performed in this study.

In this study, differentially expressed lncRNAs (DELs), miRNAs (DEMs), and mRNAs (DEGs) were obtained from TNBC samples in The Cancer Genome Atlas (TCGA) database, whereas interferon-related genes (IRGs) were downloaded from the Gene Set Enrichment Analysis (GSEA) website. Interferon-related differentially expressed mRNAs (IR-DEGs) were then obtained from the intersection between DEGs and IRGs. Next, Univariate Cox regression analysis was performed to identify IR-DEGs associated with survival. An interferon-related prognostic signature (IRPS) was further established by multivariate Cox regression analysis, and prognostic IR-DEGs (IR-DEGs-IRPS) were then identified. The abnormal expressions of these IR-DEGs-IRPS were also validated by using independent GEO cohorts. Afterward, the relationship between IRPS and immunotherapy was analyzed, revealing its immune infiltration landscape. Targeted pairing of the IR-DEGs-IRPS was performed through the Encyclopedia of RNA Interactomes (ENCORI) database to construct an interferon-related prognostic ceRNA network. The correlation between IR-DEGs expression from ceRNA and drug sensitivity was further explored using the CellMiner database. Finally, potentially significant regulatory axes within the ceRNA network were identified based on the targeted “lncRNA-miRNA-mRNA” interactions, and their potential effect in TNBC was assessed using GSEA. In addition, we validated the expression of IR-DEGs-IRPS *in vitro* (tumor versus normal cell cultures) using quantitative real-time PCR (qRT-PCR) (Figure 1). This study may offer new candidate molecular biomarkers for the poor prognosis of TNBC and contribute to a deeper understanding of the regulatory mechanisms of interferon-related ceRNA in TNBC occurrence and development.

## 2 Materials and methods

### 2.1 Data acquisition and description

RNA expression data (mRNA, lncRNA, and miRNA) associated with TNBC, along with clinical survival data, were retrieved from the TCGA database (https://portal.gdc.cancer.gov/) (Tomczak et al., 2015). Table 1 displays the sample sizes of the three kinds of RNA sequencing. A total of 464 interferon genes (Supplementary Table S1) were retrieved from the Molecular Signatures database (MSigDB) on the GSEA website (http://www.gsea-msigdb.org/gsea/index.jsp) (Subramanian et al., 2005).

**TABLE 1:** TNBC and normal samples from TCGA.

RNA sequencing	Total	Tumor	Normal
mRNA	229	116	113
lncRNA	229	116	113
miRNA	122	114	8

### 2.2 Integrative multi-omics analysis for IRPS construction and evaluation

#### 2.2.1 Acquisition of differentially expressed RNAs (DERNAs)

Differential expression analyses of mRNA, lncRNA, and miRNA from the TCGA database were performed by using the “limma” R package. Differentially expressed mRNAs (DEGs), lncRNAs (DELs), and miRNAs (DEMs) were identified based on a threshold of *P*-adj < 0.05 and |log2FC| > 1. Then, DEGs were intersected with interferon-related genes from the MSigDB database to obtain interferon-related DEGs (IR-DEGs). Heatmaps and volcano plots for IR-DEGs, DELs, and DEMs were generated using the “pheatmap” and “limma” R packages.

#### 2.2.2 Establishment of the IFN-related prognostic signature (IRPS)

The “survival” R package was used to conduct univariate Cox regression analysis on IR-DEGs, with a *P*-value <0.05 as the threshold for identifying survival-associated IR-DEGs. Multivariate Cox regression analysis was then applied to construct the interferon-related prognostic signature (IRPS). Based on the expression levels and correlation coefficients of prognostic IR-DEGs (IR-DEGs-IRPS), risk scores of the patients were calculated by the following formula: risk score = Exp (mRNA1) × β1 + Exp (mRNA2) × β2 + Exp (mRNA3) × β3… + Exp (mRNAn) × βn. Patients were divided into high- and low-risk groups according to the median risk score, and survival rates for both groups were calculated. Kaplan-Meier analysis was performed to plot the survival curves for the high- and low-risk groups, while 1-year, 3-year, and 5-year receiver operating characteristic (ROC) curves were generated to assess the reliability of the IRPS.

#### 2.2.3 External validation of prognostic gene expression using GEO datasets

To validate the expression patterns of the key genes identified from the TCGA-TNBC analysis, two independent GEO datasets, GSE65194 (GPL570; normal: TNBC = 11:41), and GSE38959 (GPL4133; normal: TNBC = 13:30), were retrieved from the GEO database (https://www.ncbi.nlm.nih.gov/gds). Gene expression matrices were log_2_-transformed where applicable. Differential expression analysis within each dataset was performed using the “limma” R package. For integrative validation, both expression matrices were merged after retaining only the intersecting genes, followed by batch effect correction using the ComBat function from the “sva” package. Statistical comparisons between normal and TNBC samples were conducted using the unmatched Wilcoxon rank-sum test, and genes with *P* < 0.05 were considered statistically significant. The validation results were visualized using box plots for each dataset and the combined batch-corrected dataset.

#### 2.2.4 Prediction of immunotherapy response in IRPS

The Tumor Immune Dysfunction and Exclusion (TIDE) algorithm integrates characteristics of T cell dysfunction and T cell exclusion to simulate tumor immune evasion based on gene expression levels, thereby predicting clinical response to immune checkpoint blockade (ICB) treatment (Jiang et al., 2018). Using the TIDE web tool (http://tide.dfci.harvard.edu/), the algorithm calculates three scores: TIDE, Exclusion, and Dysfunction. These scores represent immune evasion ability, the extent of immune cell exclusion, and the level of immune dysfunction, respectively (Fu et al., 2020). A *t*-test was performed to assess the differences in scores between high-risk and low-risk groups to evaluate potential variations in ICB response, with a significance threshold set at *P* < 0.05.

Tumor mutation burden (TMB), which represents the total number of mutations in tumor samples, is increasingly recognized as a key biomarker for assessing responses to immunotherapy (Chalmers et al., 2017). The TMB for each patient was calculated using the “maftools” package, and violin plots were generated to compare TMB across different risk groups. Spearman correlation analysis was utilized to evaluate the relationship between TMB and risk scores, with a significance threshold of *P* < 0.05.

#### 2.2.5 Analysis of tumor immune microenvironment

Single-sample gene set enrichment analysis (ssGSEA) (Hänzelmann et al., 2013) was employed to assess immune cell infiltration in TNBC. Using gene sets corresponding to 23 immune cell types identified in previous studies (Miao et al., 2020), the degree of infiltration for various immune cell types was predicted. Subsequently, Spearman correlation regression analysis was used to determine the relationships between IR-DEGs-IRPS and immune cell infiltration, which were visualized using the “ggplot2” R package.

#### 2.2.6 Construction of the ceRNA network and identification of the key regulatory axes

Relevant miRNA-mRNA pairs were extracted by the ENCORI database (http://starbase.sysu.edu.cn/) (Li et al., 2014) based on IR-DEGs-IRPS, and the miRNAs in these pairs were intersected with DEMs to identify IRPS-related DEMs. Relevant lncRNA-miRNA pairs were further extracted using the same database based on IRPS-related DEMs, and the lncRNAs in lncRNA-miRNA pairs were intersected with DELs to identify the IRPS-related DELs. The criteria for selecting upstream miRNAs that bind to mRNAs included screening at least two miRNAs from seven databases (microT, miRmap, miRanda, PicTar, PITA, RNA22, and TargetScan), while upstream lncRNAs that bind to miRNAs were screened only from the miRanda database. Then, the paired IRPS-related DELs, DEMs, and IR-DEGs were used as nodes to construct an interferon-related prognostic lncRNA-miRNA-mRNA network and were visualized by Cytoscape software.

Based on the ceRNA hypothesis, the level of lncRNA is expected to be negatively correlated with miRNA level and positively correlated with mRNA level. LncRNA can downregulate the expression level of miRNA and reduce its inhibitory effect on mRNA expression. The correlations among these three RNAs in the ceRNA network were performed using the “cor” function in R software, and the ceRNA regulatory axes were identified. A *P* < 0.05 was considered statistically significant.

#### 2.2.7 Drug sensitivity analysis

The CellMiner database (https://discover.nci.nih.gov/cellminer/home.do) (Reinhold et al., 2012) offers extensive data on gene expression and drug activity scores in tumor cells. By employing Pearson correlation analysis on the relevant drug data obtained from CellMiner, we evaluated the relationship between IR-DEGs-IRPS within ceRNAs and drug activity scores. This aimed to identify potential candidate drugs targeting IR-DEGs-IRPS in ceRNAs.

#### 2.2.8 Functional enrichment analysis

Reactome pathway gene sets (MSigDB C2: Canonical Pathways; file c2.cp.reactome.v7.5.symbols.gmt) were obtained from MSigDB and used for Gene Set Enrichment Analysis (GSEA) of IR-DEGs-IRPS genes within the ceRNA regulatory axes (Liberzon et al., 2011). We ran 1,000 permutations and considered results significant at nominal *P* < 0.05.

#### 2.2.9 Cell culture and qRT-PCR

Three TNBC tumor cell lines: MDA-MB-231 (Sangon Biotech, China), MDA-MB-468 (Sangon Biotech, China), and HCC 1806 (BDBIO, China), and normal mammary epithelial cells: MCF-10A (Sangon Biotech, China) were cultured to confluence in 6-well plates at a plating density of 50,000 cells per well. RNA extraction was conducted with Trizol reagent (Invitrogen, USA) in accordance with the established protocol. cDNA synthesis was conducted utilizing the First-Strand Synthesis Master Mix x (LABLEAD, China). Gene expression was quantified utilizing the LightCycler 480 system (Roche Life Sciences, Germany) and SYBR mixture (LABLEAD, China) with gene-specific primers (Table 2). β-Actin served as an internal reference gene for data normalization, with expression levels assessed using the 2^−ΔΔCT^ method, and results presented as a relative fold change compared to MCF-10A.

**TABLE 2:** Primers used in this study.

Gene	Primers
LAMP3	Forward:GCGTCCCTGGCCGTAATTT
	Reverse:TGCTTGCTTAGCTGGTTGCT
STXBP1	Forward:AGGGCATAACGATTGTGGAAG
	Reverse:GGAGTGATGAGATACACAGCCT
POLR2F	Forward:GCTCCAGATTGCGATGTGTG
	Reverse:GGATCTTTCGGGCCTTGAGTT
CD276	Forward:CTGGCTTTCGTGTGCTGGAGAA
	Reverse:GCTGTCAGAGTGTTTCAGAGGC

#### 2.2.10 Statistical analysis

Statistical analyses for this study were carried out using R 4.2.2, and a *P* < 0.05 was considered to indicate statistical significance.

## 3 Results

### 3.1 DELs, DEMs and IR-DEGs in TNBC

**FIGURE 2:**
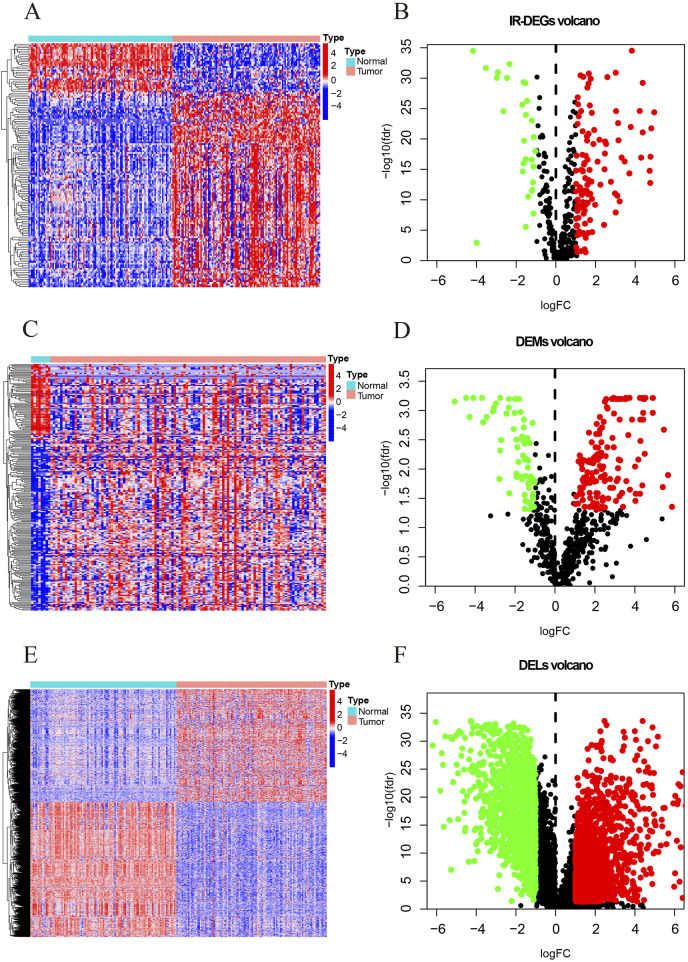
The heatmaps and volcano maps of DERNAs. **(A)** IR-DEGs heatmap. **(B)** IR-DEGs volcano maps. **(C)** DEMs heatmap. **(D)** DEMs volcano maps. **(E)** DELs heatmap. **(F)** DELs volcano maps. The red and green dots indicate DERNAs with significant upregulation and downregulation, respectively, while the black dots indicate DERNAs with no significant differences.

This study identifies a total of 139 IR-DEGs (upregulated: 111, downregulated: 28) (Figures 2A,B; Supplementary Table S2), 215 DEMs (upregulated: 148, downregulated: 67) (Figures 2C,D; Supplementary Table S3), and 1627 DELs (upregulated: 740, downregulated: 887) (Figures 2E,F; Supplementary Table S4) from TNBC samples in the TCGA dataset.

### 3.2 The establishment and evaluation of the IRPS based on IR-DEGs

The univariate Cox regression analysis identified six survival-related IR-DEGs (STXBP1, IL12RB2, CCL16, POLR2F, LAMP3, CD276) (Table 3). An IRPS for TNBC is established through multivariate Cox regression analysis, leading to the identification of four IR-DEGs-IRPS, namely, STXBP1, LAMP3, CD276, and POLR2F (Table 4). Among these, only POLR2F serves as an independent prognostic factor for TNBC, acting as a low-risk gene with a hazard ratio (HR) value of less than 1.

**TABLE 3:** The results of univariate Cox analysis.

Gene	Log_2_FC	HR	HR.95L	HR.95H	*P*-value
STXBP1	−1.535	1.182	1.047	1.334	0.007
IL12RB2	3.469	0.623	0.423	0.917	0.017
CCL16	−2.924	121.093	2.077	7058.725	0.021
POLR2F	1.093	0.021	0.001	0.589	0.023
LAMP3	3.035	0.903	0.822	0.993	0.036
CD276	1.091	1.030	1.000	1.061	0.050

**TABLE 4:** Prognostic IR-DEGs of IRPS.

Expression	Id	Coef	HR	HR.95L	HR.95H	*P*-value
Upregulated	POLR2F	−3.034	0.048	0.002	1.000	0.050
	CD276	0.024	1.025	0.996	1.054	0.094
	LAMP3	−0.078	0.925	0.840	1.018	0.110
Downregulated	STXBP1	0.109	1.115	0.979	1.270	0.101

**FIGURE 3:**
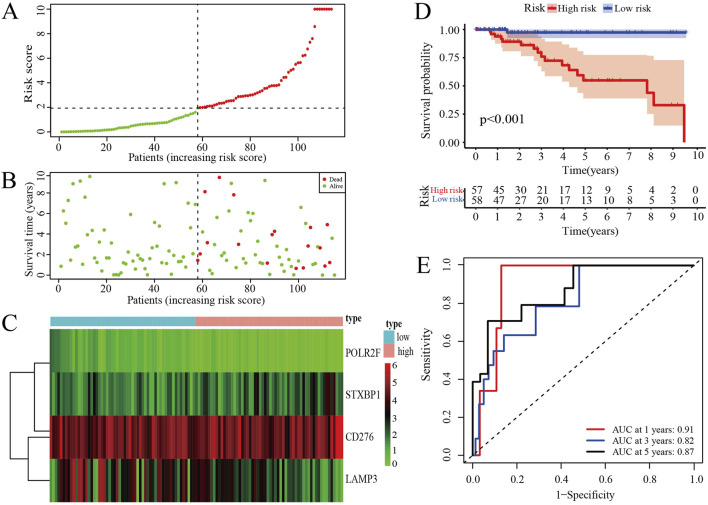
Prognosis assessments of IRPS. **(A)** Risk score curve of TNBC patients. **(B)** Survival status of TNBC patients. **(C)** The distribution of IR-DEGs-IRPS expression profiles. **(D)** Kaplan-Meier curves for high-risk and low-risk groups. **(E)** ROC curves for 1-, 3-, and 5-year survival of TNBC patients.

The risk score curve indicates that patients classified as low-risk consistently have lower risk scores, whereas those in the high-risk category exhibit substantially elevated risk scores (Figure 3A). Analysis of the survival status diagram reveals a markedly lower mortality rate for TNBC patients in the low-risk group compared to their high-risk counterparts (Figure 3B). The heatmap analysis revealed distinct expression patterns between low- and high-risk groups, with STXBP1 and CD276 showing elevated expression in high-risk patients, whereas POLR2F and LAMP3 were generally expressed at lower levels. These findings suggest that these four genes may serve as a robust prognostic signature for risk stratification (Figure 3C). Furthermore, the low-risk group is associated with a more favorable prognosis as compared with high-risk group in context of the IRPS (Figure 3D). ROC curves show that the area under the curve (AUC) values exceeds 0.8 for 1-year, 3-year, and 5-year survival, thereby confirming the strong predictive capability of IRPS for TNBC patient outcomes (Figure 3E).

### 3.3 Validation of TCGA findings using the GEO datasets

**FIGURE 4:**
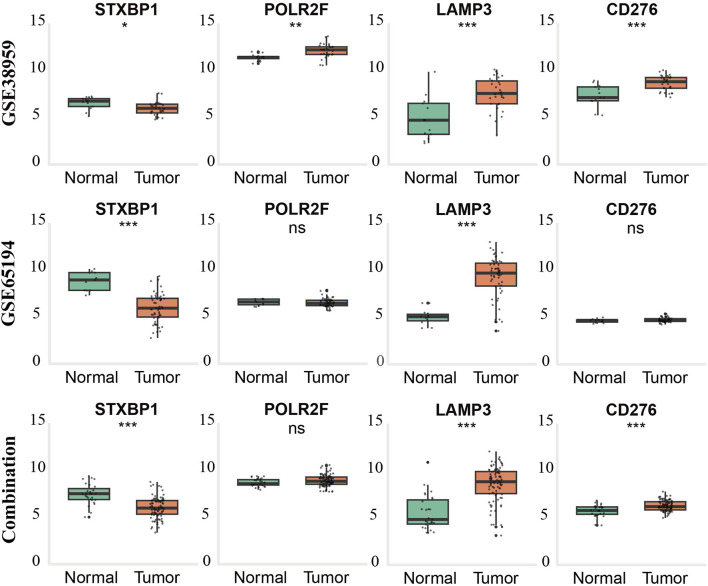
External validation of gene expression patterns in TNBC across GEO datasets and combined cohorts. Boxplots show expression levels of STXBP1, POLR2F, LAMP3, and CD276 in normal breast tissue (green) and triple-negative breast cancer (TNBC) tissue (orange) from GSE65194 (top row), GSE38959 (middle row), and the batch-effect–corrected combined dataset (bottom row). Statistical significance was determined using the Wilcoxon rank-sum test (*p* < 0.05, p < 0.01, *p* < 0.001; ns = not significant).

External validation using GSE65194, GSE38959, and a batch-corrected combined dataset confirmed consistent downregulation of STXBP1 and upregulation of LAMP3 across all datasets. CD276 and POLR2F followed the TCGA expression trends in GSE38959; however, neither gene reached statistical significance in GSE65194. In the combined dataset, CD276 remained consistent with TCGA trends, while POLR2F showed no significant difference. These results highlight complete cross-cohort reproducibility for STXBP1 and LAMP3, with acceptable reproducibility for POLR2F and CD276, as shown in Table 5 and Figure 4.

**TABLE 5:** Results of external cohort validation.

Gene	GSE65194	GSE38959	Combination	TCGA
STXBP1	▼	▼	▼	▼
POLR2F	ns	▲	ns	▲
LAMP3	▲	▲	▲	▲
CD276	ns	▲	▲	▲

Note: ▲ represents upregulated, ▼ represents downregulated and ns denotes no significant difference.

### 3.4 The relationship between IRPS and immunotherapy

**FIGURE 5:**
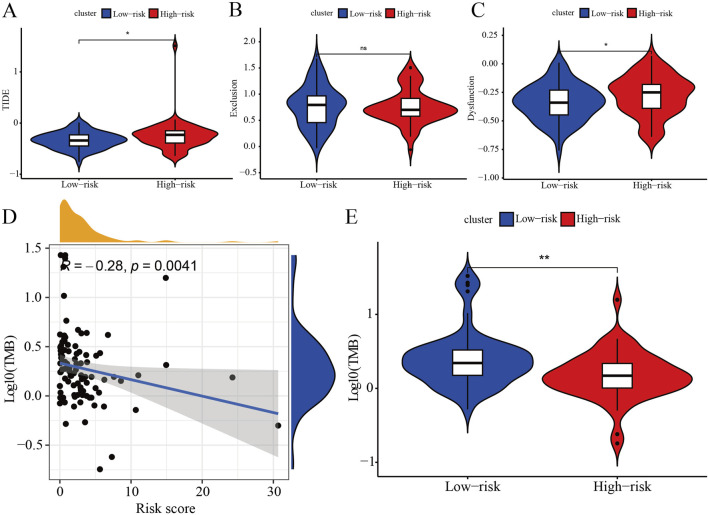
Immune assessments of IRPS. **(A)** Represents significant differences in TIDE scores between patients categorized into high-risk and low-risk groups, highlighting the observed variations and trends. **(B)** Presents a comparative analysis of Exclusion scores, emphasizing significant differences between patients classified as high-risk and those classified as low-risk. **(C)** Depicts significant differences in Dysfunction scores between patients categorized as high-risk versus low-risk, highlighting the observed variations. **(D)** Illustrates the relationship between prognostic risk scores and tumor mutation burden (TMB) scores, showing the extent of correlation between these two metrics. **(E)** Displays the significant differences in TMB scores between patients classified as high-risk and low-risk, emphasizing the observed variations.

The TIDE results reveal that both the TIDE and Dysfunction scores are significantly higher in the high-risk group compared to the low-risk group, while the Exclusion score does not differ significantly between the groups (Figures 5A–C). These findings suggest that the high-risk group exhibits higher levels of immune evasion and immune dysfunction, whereas the low-risk group may respond more favorably to ICB therapy. Correlation analysis demonstrates that the risk score is negatively linked with the TMB score (Figure 5D) and the TMB is higher in the low-risk group as compared with the high-risk group (Figure 5E). This indicates that as the risk score increases, the TMB score decreases, resulting in reduced efficacy of immunotherapy in TNBC patients. In summary, the low-risk group is likely to show a better response to immunotherapy compared to the high-risk group.

### 3.5 The connection between IRPS and immune microenvironment

**FIGURE 6:**
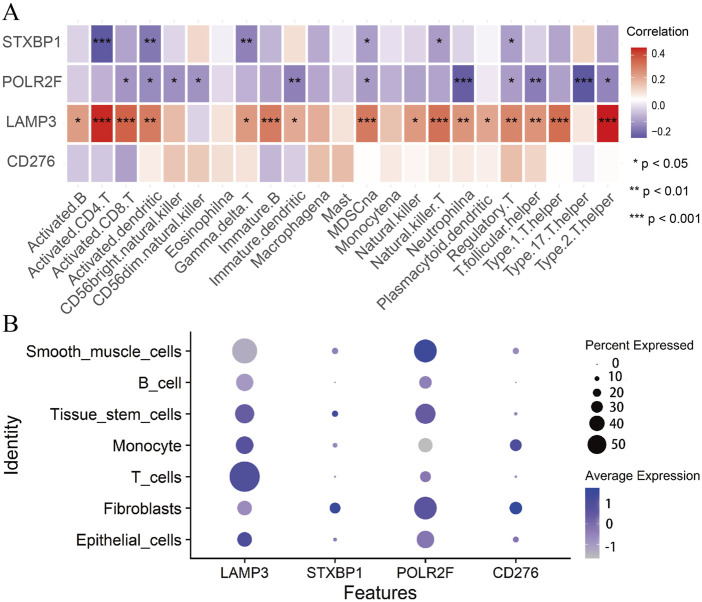
Immunocyte correlation and single-cell analysis. **(A)** The correlations between IR-DEGs-IRPS and immune cells. **(B)** Bubble plot of expression levels of IR-DEGs-IRPS in different cells.

Immunocyte correlation analysis shows that STXBP1 and POLR2F are considered immune suppressor genes. Their expressions are significantly and negatively correlated with the immune functions of several cell types, including activated CD4 T cells, neutrophils, and T helper cells 17 (Th17) (Figure 6A). In contrast, LAMP3 is regarded as an immune promoter gene, with its expression showing a significant positive correlation with the immune functions of activated CD4 T cells and T helper cells 2 (Th2) (Figure 6A). However, CD276 expression does not exhibit a significant correlation with the immune functions of 23 different immune cell types (Figure 6A). These findings indicate that IR-DEGs-IRPS are closely associated with the immune microenvironment.

Single-cell analysis shows that LAMP3 is highly expressed in T cells, tissue stem cells, monocytes, and epithelial cells (Figure 6B). In contrast, STXBP1 and POLR2F exhibit high expression levels in fibroblasts, tissue stem cells, and smooth muscle cells, while CD276 is predominantly expressed in monocytes and fibroblasts (Figure 6B). Thus, the expressions of these four IR-DEGs-IRPS in various cells are aligning well with their immunocyte correlations.

### 3.6 The construction of the lncRNA-miRNA-mRNA network associated with interferon

**FIGURE 7:**
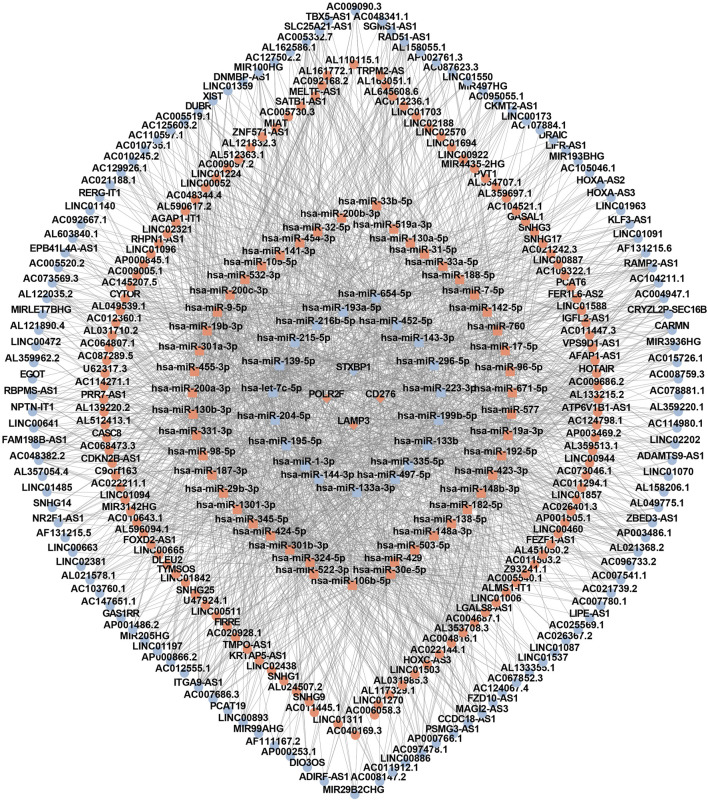
The interferon-related ceRNA network. Circles represent lncRNAs, squares represent miRNAs, and rhombuses represent mRNAs. Red denotes upregulated RNA and blue denotes downregulated RNA.

Based on four IR-DEGs-IRPS (STXBP1, LAMP3, CD276, and POLR2F), a total of 66 DEMs (Supplementary Table S5) and 248 DELs (Supplementary Table S6) were selected from the ENCORI database. As shown in Figure 7, these IR-DEGs, DEMs, and DELs constituted 38 DEM-IR-DEG pairs (Supplementary Table S7), 1798 DEL-DEM pairs (Supplementary Table S8), and 137 indirect DEL and IR-DEG pairs (Supplementary Table S9), forming an interferon-related prognostic ceRNA network.

### 3.7 Drug sensitivity analysis of IR-DEGs-IRPS

**FIGURE 8:**
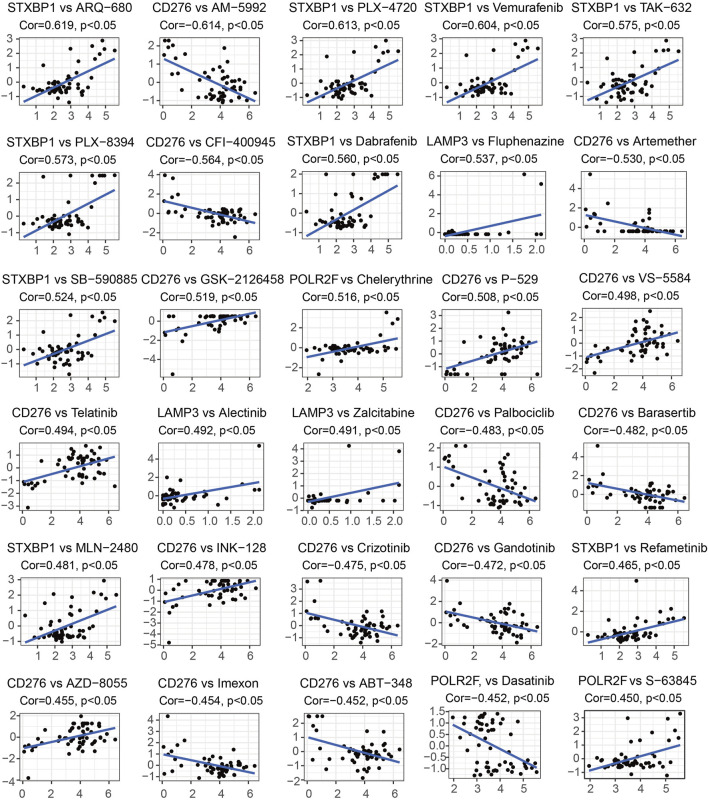
The top 30 drugs most strongly associated with IR-DEGs-IRPS. In each subfigure, the Y-axis represents the Z-score of drug activity, and the X-axis represents the expression level of IR-DEGs-IRPS. Cor stands for correlation coefficient, where its value greater than 0 indicates a positive correlation and less than 0 indicates a negative correlation.

Supplementary Table S10 displays 451 pairs between the abnormal expression of four IR-DEGs-IRPS and the drug sensitivity of 295 FDA-approved anti-tumor drugs tested in NCI-60 cancer cell lines. Among these, 126 drugs within the 155 pairs of mRNA-drug correlation pairs are found to have drug resistance due to the overexpression of IR-DEGs-IRPS, while 84 drugs in the 126 pairs exhibit the opposite effect. Furthermore, 85 drugs show opposite drug sensitivity to different IR-DEGs-IRPS across 170 pairs. Specifically, the upregulation of STXBP1 is associated with increased drug resistance to 29 drugs (ON-123300, AM-5992, TPX-0005, ARV-825, SAR-20347, etc.) and with enhanced drug sensitivity to 42 drugs (ARQ-680, PLX-4720, Vemurafenib, TAK-632, PLX-8394, etc.). The upregulation of LAMP3 is linked to increased drug resistance to 20 drugs (Sonidegib, P-529, Irofulven, PF-4989216, GDC-0084, etc.) and increased drug sensitivity to 67 drugs (Fluphenazine, Alectinib, Zalcitabine, Ribavirin, Nelarabine, etc.). The upregulation of CD276 is associated with increased drug resistance to 140 drugs (AM-5992, CFI-400945, Artemether, Palbociclib, Barasertib, etc.) and enhanced drug sensitivity to 48 drugs (GSK-2126458, P-529, VS-5584, Telatinib, INK-128, etc.). The upregulation of POLR2F is associated with increased drug resistance to 51 drugs (Dasatinib, Everolimus, LY-3023414, spebrutinib, Saracatinib, etc.), and with heightened drug sensitivity to 54 drugs (Chelerythrine, S-63845, Carmustine, auranofin, Hydroxyurea, etc.). Figure 8 presents the top 30 drugs most strongly associated with IR-DEGs-IRPS in the cancer cells. Among these, 20 drugs (ARQ-680, PLX-4720, Vemurafenib, TAK-632, PLX-8394, Dabrafenib, Fluphenazine, SB-590885, GSK-2126458, Chelerythrine, P-529, VS-5584, Telatinib, Alectinib, Zalcitabine, MLN-2480, INK-128, Refametinib, AZD-8055, S-63845) increase their drug sensitivity with the overexpression of IR-DEGs-IRPS, whereas 10 drugs (AM-5992, CFI-400945, Artemether, Palbociclib, Barasertib, Crizotinib, Gandotinib, Imexon, ABT-348, Dasatinib) exhibit increased drug resistance with the upregulated IR-DEGs-IRPS.

### 3.8 The identification of the lncRNA-miRNA-mRNA key regulatory axes

**FIGURE 9:**
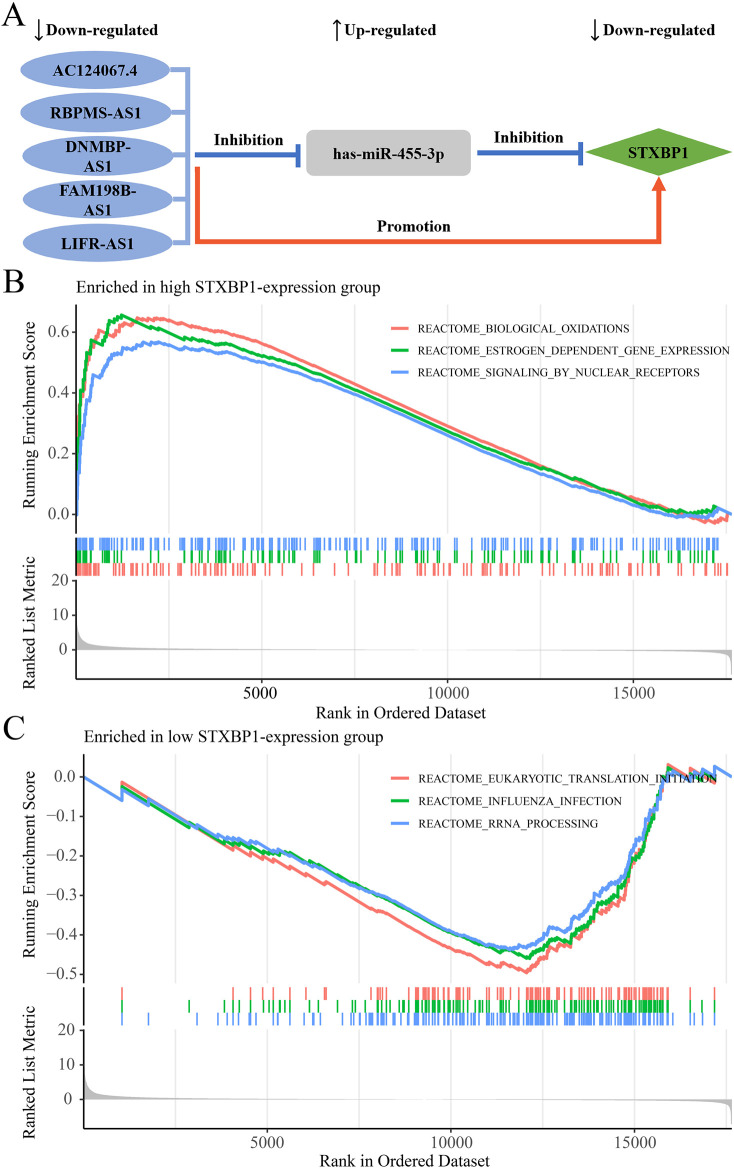
The ceRNA network regulatory axes and GSEA enrichment pathway analyses. **(A)** The targeted regulatory relationships among lncRNA, miRNA, and mRNA of five potential ceRNA axes in TNBC. **(B)** Enrichment pathways associated with STXBP1 in the high-expression group. **(C)** Enrichment pathways associated with STXBP1 in the low expression group.

In the regulatory axis, miRNA expression is negatively correlated with both mRNA and lncRNA expressions, while lncRNA expression is positively correlated with mRNA expression, consistent with the ceRNA hypothesis. Correlation analysis reveals that only hsa-miR-455-3p-STXBP1 among the 38 DEM-IR-DEG pairs showed a significant negative correlation (*P* < 0.05). Additionally, 116 of 1798 DEL-DEM pairs exhibit significant negative correlations (*P* < 0.05). Among the indirect DEL and IR-DEG pairs, 103 pairs show significant positive correlations (*P* < 0.05). By using hsa-miR-455-3p as the connecting node, eight DELs (AF111167.2, AC124067.4, RBPMS-AS1, AL359220.1, DNMBP-AS1, LINC01550, FAM198B-AS1, LIFR-AS1) negatively correlated with hsa-miR-455-3p are identified, and five of them are positively correlated with STXBP1 (Table 6). After combining these five pairs of DEL-DEM with one pair of DEM-IR-DEG, five potential lncRNA/miRNA/mRNA regulatory axes are established (AC124067.4/hsa-miR-455-3p/STXBP1, RBPMS-AS1/hsa-miR-455-3p/STXBP1, DNMBP-AS1/hsa-miR-455-3p/STXBP1, FAM198B-AS1/hsa-miR-455-3p/STXBP1, LIFR-AS1/hsa-miR-455-3p/STXBP1) (Figure 9A). Table 7 presents the differential expression of miRNA and lncRNAs within ceRNA axes in TNBC by our study.

**TABLE 6:** Correlation of DERNA pairs in the ceRNA key axes.

Derna	Derna	*r*	*P*
hsa-miR-455-3p	STXBP1	−0.20964	0.02584
hsa-miR-455-3p	AC124067.4	−0.22746	0.01540
	RBPMS-AS1	−0.20856	0.02664
	DNMBP-AS1	−0.19776	0.03577
	FAM198B-AS1	−0.19250	0.04109
	LIFR-AS1	−0.18684	0.04753
STXBP1	DNMBP-AS1	0.43245	1.72 × 10^−06^
	AC124067.4	0.23928	0.01070
	FAM198B-AS1	0.23451	0.01241
	LIFR-AS1	0.20374	0.03040
	RBPMS-AS1	0.18810	0.04603

**TABLE 7:** Differential expression of six DERNAs within ceRNA axis in TNBC in this study.

Expression	Id	Log_2_FC	*P*-value
Upregulated	hsa-miR-455-3p	3.556	1.85 × 10^−5^
Downregulated	FAM198B-AS1	−2.898	2.72 × 10^−34^
	LIFR-AS1	−1.802	1.52 × 10^−29^
	RBPMS-AS1	−1.903	2.82 × 10^−27^
	AC124067.4	−1.348	3.63 × 10^−18^
	DNMBP-AS1	−1.556	7.79 × 10^−17^

### 3.9 GSEA functional enrichment analysis

GSEA analysis reveals that, in TNBC, patients with high STXBP1 expression were predominantly enriched in 108 pathways, including those related to biological oxidations, estrogen-dependent gene expression, and signaling by nuclear receptors (Figure 9B; Supplementary Table S11). Conversely, patients with low STXBP1 expression were notably enriched in 22 pathways, including eukaryotic translation initiation, influenza infection, and RNA processing (Figure 9C; Supplementary Table S12). These findings suggest that STXBP1 may affect the occurrence and development of TNBC through these pathways.

### 3.10 Cell culture and qRT-PCR verification

**FIGURE 10:**
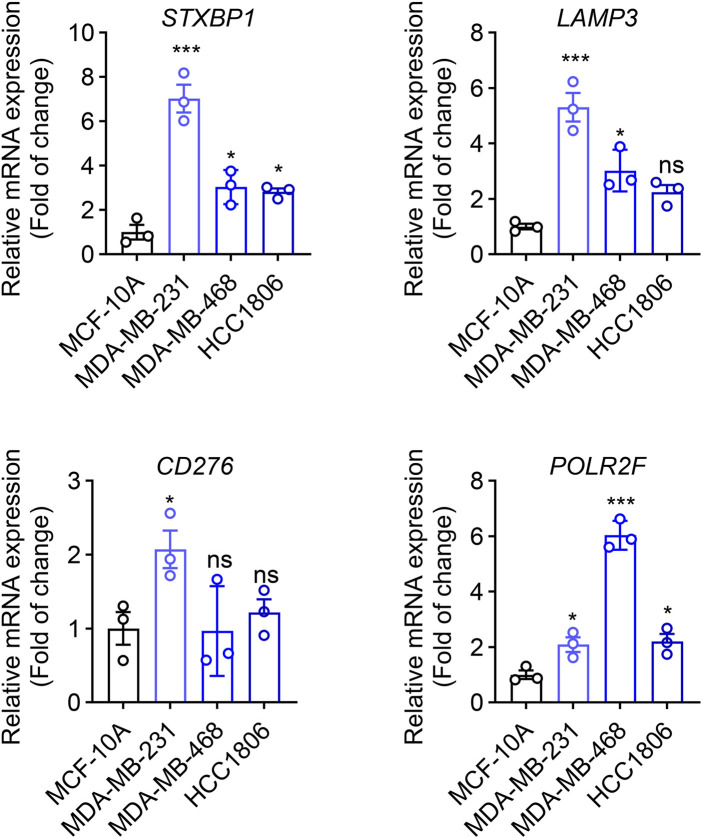
qRT-PCR analyses of four IR-DEGs-IRPS. * indicates *P* < 0.05, ** indicates *P* < 0.01, *** indicates *P* < 0.001.

The transcriptional level of four prognostic genes identified from the TNBC prognostic signature was evaluated in three TNBC tumor cell lines (MDA-MB-231, MDA-MB-468, and HCC 1806) and normal mammary epithelial cells (MCF-10A) by using qRT-PCR. As shown in Figure 10, compared with MCF-10A cells, the expressions of four prognostic genes (STXBP1, LAMP3, CD276, and POLR2F) are all significantly upregulated in MDA-MB-231 cells (*P* < 0.05). In MDA-MB-468 cells, the expression levels of three prognostic genes, STXBP1, LAMP3, and POLR2F, were significantly upregulated (*P* < 0.05). In HCC1806 cells, STXBP1 and POLR2F showed significant upregulation (*P* < 0.05). Although LAMP3 and CD276 in HCC1806 also exhibited upward trends, these changes did not reach statistical significance (Figure 10). Overall, qRT-PCR results showed that all four prognostic mRNAs were upregulated in TNBC cells.

## 4 Discussion

Compared with other breast cancer subtypes, chemotherapy remains the primary treatment for triple-negative breast cancer (TNBC) due to its aggressive nature and poor prognosis, and there are no other approved targeted therapies (Chaudhary et al., 2018). IFN plays a vital role in anti-tumor immunity (Thibaut et al., 2020). LncRNA can act as ceRNAs for miRNAs, thereby targeting and regulating mRNAs and forming lncRNA-miRNA-mRNA regulatory axes. This process participates in the occurrence and development of TNBC (Alkan and Cansaran-Duman, 2025). Therefore, studying the construction of interferon-related ceRNA networks is beneficial for understanding the molecular mechanisms of interferon genes in TNBC and for identifying new prognostic biomarkers and therapeutic targets for TNBC.

Our study identified DERNAs comprising 139 IR-DEGs, 215 DEMs, and 1627 DELs based on TNBC RNA profiles from the TCGA database and the interferon gene set from the GSEA website. The IRPS, consisting of CD276, POLR2F, LAMP3, and STXBP1 genes, was established through univariate and multivariate Cox regression analyses. Table 8 presents the aberrant expressions of these four IR-DEGs-IRPS in TNBC compared to the corresponding mRNAs reported in existing literature.

**TABLE 8:** Comparison of the aberrant changes of IR-DEGs-IRPS expressions in this study with previous TNBC literature.

IR-DEGs-IRPS	Feature	qRT-PCR	Abstract
CD276	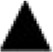	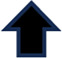	Shi et al. (2025) revealed through *in vitro* and *in vivo* experiments that CD276 is highly expressed in TNBC tumor cells under the regulation of UBE2T/CDC42/CD276 axis, and its upregulation damages the function of CD8^+^ T cells, leading to tumor cell immune escape and brain metastasis
LAMP3	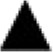	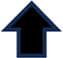	Nagelkerke et al. (2011) detected by real-time polymerase chain reaction and immunohistochemistry that LAMP3 is highly expressed in TNBC cells, and its overexpression is associated with hypoxia-induced treatment resistance and tumor metastasis
POLR2F	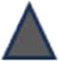	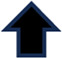	Utilizing TCGA and Gene Expression Omnibus (GEO) databases, Naorem et al. (2019) discovered that POLR2F is overexpressed in TNBC samples and is identified as an oncogene
STXBP1	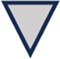	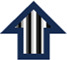	By studying the data of breast cancer patients in UCSC database and TCGA database, Mao et al. (2024) determined that STXBP1 is lowly expressed in breast cancer tissues and is a prognostic characteristic gene of breast cancer

Notes: 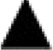 According to previous experimental reports, IR-DEG_-IRPS_ was upregulated in TNBC, aligning with our computational results.

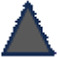
 According to previous calculation reports, IR-DEG_-IRPS_ was upregulated in TNBC, aligning with our computational results.

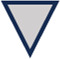
 According to previous calculation reports, IR-DEG_-IRPS_ was downregulated in BC, aligning with our computational results in TNBC.

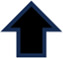
 According to previous experimental reports, IR-DEG_-IRPS_ was upregulated in TNBC, aligning with our qRT-PCR results.

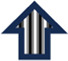
 According to previous calculation reports, IR-DEG_-IRPS_ was downregulated in BC, inconsistent with our qRT-PCR results in TNBC.

Literature evidence supports the overexpression of key genes such as CD276, LAMP3, and POLR2F in TNBC, consistent with our computational analyses from TCGA and qRT-PCR validation. This pattern was further reinforced through external validation in two independent GEO datasets (GSE65194 and GSE38959) and in a merged cohort following rigorous batch effect correction, where LAMP3, CD276 and POLR2F demonstrated particularly consistent upregulation across datasets. In contrast, STXBP1 has been reported as downregulated in breast cancer in prior bioinformatics studies, which is in agreement with our *in silico* analyses from TCGA and GEO but differs from our qRT-PCR findings in TNBC cell lines. Such discrepancies between *in silico* and *in vitro* results are well-documented and are often driven by biological heterogeneity rather than technical artifacts. Bulk RNA-seq data from TCGA capture an admixture of tumor, stromal, and immune cells, along with inter-patient variability in tumor purity, treatment history, and disease stage, all of which can influence gene-level expression estimates (Turashvili and Brogi, 2017; Yadav and De, 2015). Conversely, qRT-PCR assays in homogeneous *in vitro* cell populations lack the complex tumor microenvironment and cell–cell interactions present *in vivo*, which can yield divergent expression patterns (Yu et al., 2019). The consistent validation of most target genes across independent cohorts nevertheless underscores the robustness and generalizability of our findings.

The inclusion of LAMP3, POLR2F, and STXBP1 in the IRPS reflects their complementary immune-modulatory functions and consistent prognostic association in TNBC. LAMP3, predominantly expressed in tumor-associated dendritic and myeloid cells, promotes antigen presentation and modulates T-cell activation (Dong et al., 2025; Li et al., 2023). POLR2F, a subunit of RNA polymerase II, has been implicated in DOK3-mediated TNF/MAPK signaling, influencing macrophage recruitment and polarization (Li P. et al., 2022). STXBP1, a regulator of vesicle docking and fusion, helps CXCL chemokine release, promoting macrophage infiltration and immune evasion (Hao et al., 2025). These mechanisms span antigen presentation, inflammatory transcriptional control, and chemokine-mediated immune cell trafficking, three distinct but converging aspects of tumor–immune crosstalk. Multivariate analyses confirmed that specific co-expression patterns of these genes were significantly associated with TNBC survival and recurrence, supporting their combined prognostic value even in the absence of demonstrated direct physical interactions. We acknowledge that functional relationships among the three genes have not yet been experimentally validated; targeted perturbation and co-culture studies will be an important direction for future work. CD276, which emerged from the same regression modelling process, was also evaluated alongside these genes for prognostic and immunological relevance.

Immunotherapy is becoming increasingly recognized as a strategic approach to cancer treatment (Waldman et al., 2020). As a significant component of immunotherapy, interferon can elicit a robust immune response during anti-tumor processes (Girard et al., 2025). TMB and TIDE are two common methods for assessing the efficacy of immunotherapy. Patients with high TMB scores will benefit from immunotherapy, for example, patients with mismatch repair deficiency colorectal cancer (having a large number of somatic mutations) had better immune-related progression-free survivals on PD-1/PD-L1 antibody therapy (Le et al., 2015). TIDE improves the prediction of immune checkpoint inhibitor efficacy by integrating two mechanisms of tumor immune escape (immune rejection and immune dysfunction) rather than relying on a single biomarker (Le et al., 2015). Considering these, we assessed the relevance of the IRPS risk score and immunotherapy. Our analysis revealed that TNBC patients with low-risk scores had a higher tumor mutation burden and a lower level of immune escape, suggesting they may benefit more from immunotherapy. Early-stage low-risk TNBC with strong immunogenicity exhibits higher levels of TMB, PD-L1 expression, and tumor-infiltrating lymphocytes (TILs), which contribute to enhanced immunologic function and better response to immunotherapy (Geurts and Kok, 2023; Keenan and Tolaney, 2020). Our findings emphasize the beneficial role of IRPS in future immunotherapy strategies for TNBC.

Interestingly, our immune cell infiltration analyses revealed that STXBP1 and POLR2F exhibited significant negative correlations with the infiltrating abundance of various immune cells, including dendritic cells, γδT cells, myeloid-derived suppressor cells (MDSC), natural killer T cells, and regulatory T cells. Previous research has described that IFN-γ expression is positively linked with dendritic cells (Xu et al., 2021) and γδT cells (Yan et al., 2025), as well as negatively regulating the production of regulatory T cells (Troschke-Meurer et al., 2019). In turn, regulatory T cells can directly or indirectly suppress the proliferation and function of CD4 and CD8 T cells, B cells, dendritic cells, macrophages, and natural killer cells (McCallion et al., 2023). These findings suggest that STXBP1 and POLR2F may inhibit the tumor immune microenvironment through IFN-γ-mediated pathways. Moreover, a significant positive correlation is detected between LAMP3 and immune cell infiltration, including CD4 T cell, CD8 T cell, natural killer T cell, and Th2 cell. Studies have demonstrated that IFN-λ expression is positively correlated with CD4 T cells, CD8 T cells, and natural killer T cells (Fu et al., 2020; Weir et al., 2023), and its overexpression can accelerate the differentiation of Th1 cells while inhibiting Th2 cell-mediated reactions (Koltsida et al., 2011). Additionally, it is widely accepted that Th2 cytokines can promote tumor progression (Jou, 2023). These findings imply that LAMP3 may contribute to tumor progression through IFN-λ-mediated pathways.

According to the results of single-cell analyses, LAMP3, identified as a critical immune-promoting gene, shows high expression in T cells, monocytes, tissue stem cells, and epithelial cells based on single-cell RNA sequencing (scRNA-seq) data. Notably, LAMP3 expression is most prominent in T cells, and T cells are associated with a better prognosis of BC (Stanton and Disis, 2016). This suggests that LAMP3 may be relevant to good survival outcomes in TNBC patients. In contrast, STXBP1 and POLR2F, both categorized as immune-suppressing genes, exhibit high expression levels in fibroblasts, tissue stem cells, and smooth muscle cells in scRNA-seq data. This aligns with prior research indicating that fibroblasts and associated stromal cells contribute to immune evasion in the TME by activating cytokine profiles and reducing T cell activity (Koppensteiner et al., 2022). CD276 exhibits predominant expression in monocytes and fibroblasts. CD276 may operate through alternative pathways, potentially involving stromal-immune interactions or modulation of the extracellular matrix, thereby indirectly influencing immune cell behavior. The single-cell sequencing results from our study offer a nuanced perspective on the immune landscape within the TME, highlighting the diverse roles of immune-regulatory genes in different cellular contexts. Immune-activating genes are upregulated in pro-immune cells, while immunosuppressive genes are upregulated in anti-immune cells, and LAMP3, STXBP1, and POLR2F conform to this expression pattern. This consistency allows these three genes to serve as potential biomarkers or therapeutic targets, particularly in the context of personalized cancer immunotherapy. While our single-cell RNA-seq analysis provides insight into the spatial and cellular expression of LAMP3 and CD276, we acknowledge that these findings are correlative. However, previous studies have demonstrated that LAMP3^+^ dendritic cells can drive T cell exhaustion and Treg recruitment (Wang et al., 2024), and that CD276 suppresses T cell infiltration and modulates macrophage polarization in tumors (Liu et al., 2024), suggesting potential causative roles for these genes in immune modulation. Nonetheless, functional validation is required to confirm these mechanisms in TNBC.

Another important finding of this study is the construction of an interferon-related prognostic ceRNA network consisting of 248 lncRNAs, 66 miRNAs, and 4 mRNAs based on prognostic IR-DEGs. Five key regulatory axes (AC124067.4/hsa-miR-455-3p/STXBP1, RBPMS-AS1/hsa-miR-455-3p/STXBP1, DNMBP-AS1/hsa-miR-455-3p/STXBP1, FAM198B-AS1/hsa-miR-455-3p/STXBP1, LIFR-AS1/hsa-miR-455-3p/STXBP1) were identified. Notably, these five ceRNA axes have not been previously reported in any studies. However, the aberrant changes of these axis-associated RNA expressions were demonstrated by previous cancer studies (Table 9). The overexpression of miRNA (hsa-miR-455-3p) of the ceRNA regulatory axes in TNBC has been confirmed by previous experimental studies, aligning with our results. Among the five lncRNAs in the key axes, the downregulation of DNMBP-AS1 and LIVR-AS1 has been experimentally or bioinformatically proven by previous studies. In addition, AC124067.4 is shown to be upregulated in the other bioinformatics study of breast cancer, which contradicts our finding for TNBC. To investigate this discrepancy further, we conducted differential expression analysis of breast cancer data based on the TCGA database and discovered that AC124067.4 is downregulated in breast cancer, though without statistical significance (log2FC = −0.536, *P* = 0.738). We believe that the differences may arise from variations in processing methods or samples included in the two studies. These findings support the reliability of ceRNA regulatory axes to a certain degree. Notably, the abnormal expression of RBPMS-AS1 and FAM198B-AS1 has not been previously documented in TNBC, and their identification highlights their potential as novel cancer biomarkers, warranting further investigation.

**TABLE 9:** Comparison of the aberrant expressions of six DERNAs in the ceRNA axes with previous TNBC literature.

Derna	Feature	Abstract
hsa-miR-455-3p	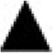	Li et al. (2017) found through qRT-PCR analysis that miR-455-3p levels are significantly increased in TNBC cells. They also demonstrated that miR-455-3p promotes tumor cell invasion and migration by targeting the tumor suppressor EI24, suggesting that miR-455-3p may serve as a potential prognostic biomarker and therapeutic target in TNBC (Li et al., 2017)
DNMBP-AS1	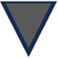	According to bioinformatics analysis of the TCGA database and qRT-PCR experiments, Gao et al. (2021) reported that the expression level of DNMBP-AS1 is decreased in BC cells and tissues, whereas the knockdown of DNMBP-AS1 is significantly correlated with a lower overall survival rate in BC patients
LIFR-AS1	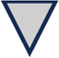	By constructing lncRNA-mediated cross-talk pathway networks in breast cancer subtypes, Wang et al. (2016) identified that LIPR-AS1 is lowly expressed in BC tissues and is involved in the regulation of proliferation, differentiation, and apoptosis of the tumor
AC124067.4	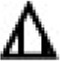	Through bioinformatics analysis of the TCGA database, Dai et al. (2022) illustrated that AC124067.4 is overexpressed in BC (as shown in Supplementary Table S1 of their article) and is an immune-related prognostic biomarker for BC
RBPMS-AS1 FAM198B-AS1	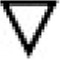	In this study, RBPMS-AS1 and FAM198B-AS1 are downregulated in TNBC. This is the first time that these two lncRNAs have been found to be abnormally expressed in TNBC and is a new biomarker for this tumor

Notes: 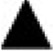 As reported experimentally, RNA was upregulated in TNBC, consistent with our computational result.

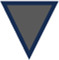
 As reported experimentally, RNA was downregulated in BC, consistent with our computational result in TNBC.

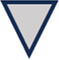
 As reported computationally, RNA was downregulated in BC, consistent with our computational result in TNBC.

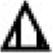
 As reported computationally, RNA was upregulated in BC, inconsistent with our computational result.

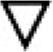
 In our prediction, RNA was downregulated in TNBC, and its abnormal expression in breast cancer had not been reported.

Through drug sensitivity analysis, 295 FDA-approved anti-tumor drugs are screened based on the altered expressions of IR-DEGs-IRPS within the ceRNA network. This investigation reveals that 126 drugs show an increase in drug resistance, while 84 drugs exhibit an enhancement in drug sensitivity correlated with the overexpression of interferon-related mRNAs. These findings potentially offer better clinical treatment options for TNBC patients. Among the top 30 mRNA-drug pairs with the highest correlation, the mechanisms of action for several drugs have been confirmed by previous studies, such as Fluphenazine and GSK-2126458. The drug sensitivity of Fluphenazine increased with the upregulation of LAMP3 expression. Xu et al. (2019) found that Fluphenazine can restrict the growth of TNBC cells and promote cell apoptosis by inhibiting the expression of ERK and AKT in RAS/RAF/MEK/ERK and PI3K-AKT-mTOR pathways. Sun et al. (2022) demonstrated that ERK and AKT can be activated by overexpression of LAMP3, thereby promoting Kaposi sarcoma-associated herpes virus cleavage, replication, and virion production. Therefore, in order to better exert the anti-tumor effect of Fluphenazine in TNBC patients, the specific mechanism of the interaction between LAMP3 and ERK or AKT in TNBC needs further investigation. Similarly, the drug sensitivity of GSK-2126458 increased with the upregulation of CD276. Leung et al. (2014) illustrated that GSK-2126458 plays an anti-tumor role in TNBC by inhibiting the PI3K/AKT/mTOR signaling pathway. CD276 has been shown to activate HIF-1α, as discovered by Hu et al. (2024), further promoting the overexpression of HB-EGF, and ultimately stimulating the proliferation, invasion, and angiogenesis of colorectal cancer cells. This suggests that understanding the interaction mechanism between CD276 and the PI3K/AKT/mTOR pathway in TNBC development will enhance the therapeutic value of GSK-2126458. However, other drugs, including ARQ-680, AM-5992, PLX-4720, TAK-632, and PLX-8394, have not been reported for TNBC therapy. Therefore, the therapeutic effects and mechanisms of these drugs on TNBC remain to be further explored. These findings can assist clinicians in selecting more sensitive and precise drugs for individual TNBC patients with aberrant expression of different prognostic genes. Thus, our study provides a new perspective for the development of precision medicine and serves as a reference for research related to chemotherapy efficacy in TNBC patients.

Interferon plays a crucial role in the tumor microenvironment of solid tumors (Martini et al., 2023). Understanding how interferon genes influence the molecular mechanisms of cancer is essential for advancing cancer treatment (Duplisea et al., 2019). The characteristics of interferon genes can make them reliable biomarkers for predicting prognosis and treatment response in TNBC. Moreover, further exploration of the underlying mechanisms of the interferon-related ceRNA regulatory axis in cancer progression is of great significance. At present, the mechanisms of ceRNA related to TNBC and interferon are not well understood. The ceRNA regulatory axes established in this study based on interferon genes can provide a theoretical framework for understanding the progression of TNBC and exhibit new prospects and candidate therapeutic targets for TNBC treatment.

Despite the promising findings, several limitations should be acknowledged. First, the prognostic signature and ceRNA network were derived from retrospective public data (TCGA), which may introduce biases related to sampling, processing, and inter-patient heterogeneity; prospective validation in real-world TNBC cohorts is warranted. Second, the interferon-related ceRNA axes were inferred computationally; the proposed lncRNA-miRNA-mRNA relationships remain hypothetical and require experimental confirmation (e.g., dual-luciferase reporter assays, gain-/loss-of-function, and rescue experiments). Third, to construct the ceRNA network, we relied primarily on miRanda for lncRNA-miRNA predictions; we recognize this may permit false positives, future work will incorporate weighted evidence frameworks and targeted experimental validation. Fourth, all five identified axes share the same miRNA–mRNA pair (hsa-miR-455-3p/STXBP1), which may reflect analytical redundancy or network bias rather than distinct biology; these findings should be interpreted with caution until validated. Fifth, our qRT-PCR validation used a limited panel of TNBC cell lines (MDA-MB-231, MDA-MB-468, and HCC 1806) and a non-tumorigenic control (MCF-10A). Although HCC1806 was added to introduce a distinct mutational background, this panel does not capture the full molecular and clinical heterogeneity of TNBC, and tissue-level validation (e.g., human TNBC vs matched normal, with protein assays such as Western blot/IHC/ISH) was not performed. Sixth, single-cell and immune-infiltration analyses are correlative and do not establish causality; perturbation studies (gene editing, *in vivo* models, or blockade experiments) are needed to determine direct effects on tumor–immune interactions. Finally, drug-sensitivity findings were based on correlations from CellMiner/NCI-60, which may not reflect TNBC complexity; pharmacologic testing in TNBC-matched models (e.g., organoids, PDX) and integration with TNBC-specific pharmacogenomic datasets are needed to enhance translational relevance.

## 5 Conclusion

In summary, this study constructed a prognostic signature and a ceRNA network associated with interferon for TNBC through data mining and bioinformatics analysis. Five ceRNA regulatory axes (AC124067.4/hsa-miR-455-3p/STXBP1, RBPMS-AS1/hsa-miR-455-3p/STXBP1, DNMBP-AS1/hsa-miR-455-3p/STXBP1, FAM198B-AS1/hsa-miR-455-3p/STXBP1, LIFR-AS1/hsa-miR-455-3p/STXBP1) were identified. Additionally, we discovered that STXBP1 and two downregulated lncRNAs (RBPMS-AS1 and FAM198B-AS1) serve as novel molecular biomarkers for TNBC. These findings may significantly contribute to our understanding of the occurrence and development of TNBC.

## Data Availability

The datasets and resources used in this study are publicly available, and all relevant details, including database links and accession numbers, are provided within the article. Supplementary Tables supporting the findings of this study are available in the Supplementary Material.
